# Effect of prednisolone on inflammatory markers in pericardial tuberculosis: A pilot study

**DOI:** 10.1016/j.ijcha.2017.10.002

**Published:** 2017-11-03

**Authors:** Justin Shenje, Rachel P. Lai, Ian L. Ross, Bongani M. Mayosi, Robert J. Wilkinson, Mpiko Ntsekhe, Katalin A. Wilkinson

**Affiliations:** a The Cardiac Clinic, Department of Medicine, University of Cape Town, Groote Schuur Hospital, Observatory 7925, South Africa; b The Francis Crick Institute, NW1 2AT London, United Kingdom; c Wellcome Centre for Infectious Diseases Research in Africa, Institute of Infectious Disease and Molecular Medicine, University of Cape Town, Observatory 7925, South Africa; d Imperial College London, W2 1PG, United Kingdom

**Keywords:** Tuberculosis, HIV, Pericarditis, Steroids, Treatment monitoring

## Abstract

**Background:**

Pericardial disorders are a common cause of heart disease, and the most common cause of pericarditis in developing countries is tuberculous (TB) pericarditis. It has been shown that prednisolone added to standard anti-TB therapy leads to a lower rate of constrictive pericarditis. We conducted a pilot study to evaluate the effect of adjunctive prednisolone treatment on the concentration of inflammatory markers in pericardial tuberculosis, in order to inform immunological mechanisms at the disease site.

**Methods:**

Pericardial fluid, plasma and saliva samples were collected from fourteen patients with pericardial tuberculosis, at multiple time points. Inflammatory markers were measured using multiplex luminex analysis and ELISA.

**Results:**

In samples from 14 patients we confirmed a strongly compartmentalized immune response at the disease site and found that prednisolone significantly reduced IL-6 concentrations in plasma by 8 hours of treatment, IL-1beta concentrations in saliva, as well as IL-8 concentrations in both pericardial fluid and saliva by 24 hours.

**Conclusion:**

Monitoring the early effect of adjunctive immunotherapy in plasma or saliva is a possibility in pericarditis.

## 1 Introduction

Tuberculosis (TB) is the most common opportunistic infection in HIV-1 infected persons in Sub-Saharan Africa. Tuberculous pericarditis is an inflammation of the pericardium caused by *Mycobacterium tuberculosis*. It is a life-threatening extrapulmonary form of TB that results in accumulation of fluid around the heart, potentially leading to constriction. In developed countries it accounts for ~ 5% of all cases of acute pericarditis, compared to up to 90% in Sub-Saharan Africa, where it is the most common cause of pericardial effusions in HIV-1 co-infected patients [1]. Mortality in these patients is up to 40% in the absence of anti-retroviral treatment, despite antituberculosis therapy, pericardial drainage, or pericardiectomy [2].

The recently completed Investigation of the Management of Pericarditis (IMPI), a large clinical trial examining the effect of prednisolone or *Mycobacterium indicus pranii* or both added to the standard regimen of isoniazid, rifampin, ethambutol and pyrazinamide showed, that neither the standard anti-TB therapy alone nor the addition of prednisolone to chemotherapy resulted in a clinically satisfactory mortality reduction, however the prednisolone group had a lower rate of constrictive pericarditis and fewer hospitalisations compared to placebo [3]. Since we have recently shown that pericardial tuberculosis is characterised by a compartmentalized profibrotic immune response, we hypothesised that prednisolone had a suppressive effect on the concentration of inflammatory and potentially profibrotic cytokines in the pericardium [4].

In this pilot study we evaluated the effect of prednisolone on inflammatory markers in pericardial fluid, plasma and saliva, in a subset of patients from the above clinical trial in order to improve our understanding of immunological mechanisms at the disease site, which could inform development of more targeted interventions. Specific analytes were selected based on our recent analysis of differentially abundant inflammatory markers (at both RNA and protein level) between the blood and pericardial fluid compartments [4]. Additionally, we also evaluated the hypothesis that inflammation induced cell death in the pericardial compartment would be in part due to apoptosis, which is initiated by two major pathways: the extrinsic (through ligation of death receptors or TNF receptors) and intrinsic pathways (mitochondria mediated) [5]. Caspase 8 activation is an essential early step in the induction of the apoptosis by the extrinsic pathway, while Caspase 9 activation is part of the intrinsic pathway, both leading to activation of Caspase 3 [6]. We therefore also assessed the effect of prednisolone on the concentration of Caspases 3, 8 and 9 in the pericardial fluid and plasma.

## 2 Materials and methods

### 2.1 Patient population

Patients were recruited from Groote Schuur Hospital, Cape Town, South Africa as part of an intensive pharmacokinetic sampling study of the Investigation of the Management of Pericarditis (IMPI) trial, with a computer-generated randomisation list as described previously [3], [7]. Ethical approval for these studies was obtained from the Faculty of Health Sciences Human Research Ethical Committee at the University of Cape Town (Reference numbers 102/2003, 402/2005, 289/2007) and as published [3], [7]. Briefly, patients were eligible for inclusion in the trial if they were 18 years of age or older, had a pericardial effusion requiring pericardiocentesis confirmed by echocardiography, had evidence of definite or probable tuberculous pericarditis and started antituberculosis treatment less than a week before enrolment into the comparison of prednisolone versus placebo arm of the IMPI trial and provided written informed consent for inclusion in the study. Pericardial fluid, plasma and saliva were obtained from 14 patients at multiple time points following randomisation to prednisolone/placebo for pharmacokinetic studies to assess antibiotic penetration as described [7]. Pericardial samples were collected via a catheter which was left in the pericardial space for 24 h post-pericardiocentesis. Not all samples were available from all patients at all time points, thus the present analysis evaluated the concentration of cytokines, chemokines and caspases at 0, 8 and 24 h only.

### 2.2 Luminex multiplex assay for cytokines and chemokines

Mediators analysed in undiluted plasma (P), pericardial fluid (PF) and saliva (S) samples included IFN-gamma, IL-1alpha, IL-1beta, IL-6, IL-10, IL-12p40, TNF, CXCL8 (IL-8) and CXCL10 (IP-10), using customized Milliplex™ kits (HCYTOMAG-60 K, Millipore, St Charles, MO, USA) on the Bio-Plex platform (Bio-Rad Laboratories, Hercules, CA, USA) as described [8]. Caspases 3, 8 and 9 in undiluted pericardial fluid and plasma were measured using human in vitro ELISA kits from Abcam (Cambridge, UK), following the manufacturer's recommendations.

### 2.3 Statistical analysis

Statistical analysis was performed using GraphPad Prism Version 7.0c for Mac. The normality of data was assessed using the D' Agostino and Pearson normality test. As not all patients were sampled at all time points, some data were not paired. Between group comparisons of unpaired non-normally distributed data were analysed using the Mann-Whitney *U* test. No correction for multiple comparisons was performed.

## 3 Results

Of the 14 patients nine were on placebo and five on prednisolone (at a dose of 120 mg per day in the first week). Patient characteristics were previously described as part of the main study [7], with the subset included in this pilot study being detailed in Table 1. Nine patients had definite tuberculous pericarditis (3 on prednisolone and 6 on placebo) and five had probable tuberculous pericarditis (2 on prednisolone and 3 on placebo). The pericardial protein measured in pericardial fluid was median 61 g/l (IQR 55–67.5) in the definite TB patients and median 66 g/l (IQR 56–76) in the probable TB patients respectively, while the ADA was median 87.3 U/l (IQR 43–127) and 51 U/l (32.8–67.6) in the definite and probable patients respectively. There was no significant difference between the groups with respect to either parameter. There was no difference between age and weight between the groups, gender composition was male/female 5/4 and 2/3 for the placebo and prednisolone groups respectively. Seven out of nine patients in the placebo and three out of five patients in the prednisolone group were HIV infected. Only two patients were receiving antiretroviral treatment at the time of the study, both in the placebo group (Table 1). Median CD4 counts were not different between the groups (240 cells/ml for the placebo and 139 cells/ml for the prednisolone group). Since our previous findings indicated that neither HIV-1 coinfection status, nor CD4 count or pericardial fluid *Mycobacterium tuberculosis* culture result affected the compartmentalized profibrotic immune response [4], we combined patients with and without HIV infection for analysis, as well as patients with definite and probable pericarditis.

**Table 1:** Patient characteristics.

Patient number	Age (years)	Wt (kg)	Gender	HIV	HAART	ART therapy	CD4 (cells/μl)	Creatinine (μmol/l)	Globulin (g/l)	Steroid allocation	Pericardial protein (g/l)	ADA (units/l)	TB microscopy
1	28	53	male	Positive	No	N/A	115	76	46	Placebo	58	Hemolyzed	Positive
2	29	40	female	Positive	No	N/A	50	20	45	Placebo	54	83	Positive
3	24	66	female	Positive	No	N/A	42	43	56	Prednisolone	68	57	Positive
4	56	82	male	Negative	N/A	N/A	485	121	30	Placebo	62	133	Positive
5	31	72	male	Negative	N/A	N/A	319	82	36	Prednisolone	55	119	Positive
6	51	53	female	Positive	Yes	Tdf/FTC/EFV	159	80	56	Placebo	50	53	Negative
7	24	45	female	Positive	No	N/A	321	257	56	Placebo	67	92	Positive
8	27	66	female	Positive	No	N/A	135	45	55	Prednisolone	70	26	Positive
9	44	52	female	Positive	No	N/A	139	65	56	Prednisolone	66	51	Negative
10	59	66	male	Negative	N/A	N/A	874	97	38	Prednisolone	62	33	Negative
11	45	70	male	Negative	N/A	N/A	721	109	47	Placebo	76	32	Negative
12	33	47	male	Positive	No	N/A	116	71	49	Placebo	61	130	Positive
13	25	73	male	Positive	No	N/A	a	73	a	Placebo	55	38	Positive
14	27	a	female	Positive	Yes	Tdf/FTC/EFV	255	41	74	Placebo	76	68	Negative

a Data not available.

Results are summarised in Table 2, indicating the median (IQR) of all analytes measured in all samples at the tested time points. Since samples were collected before prednisolone administration, we combined the baseline data to analyse the effect of compartmentalization: higher concentrations of IFN-gamma, IL-10, IL-1beta, IL-6, IL-8, IP-10 and TNF were found at the disease site compared to plasma at day 0, supporting our previous findings [4]. Interestingly, IL-1alpha and IL-12p40 was mostly detectable in the saliva samples, which also contained elevated concentrations of IL-1beta, IL-8 and IP-10 compared to plasma. Only IL-6 concentrations were higher in the plasma, compared to saliva (p = 0.03) at baseline. Caspase 3, 8 and 9 were only detectable in the pericardial fluid as opposed to plasma (p = 0.02, 0.0001 and 0.03 respectively for the three caspases measured).

**Table 2:** Median (IQR) of all analytes measured in all samples.

Site	N	Time	Group	IFN-γ pg/ml	IL-1α pg/ml	IL-1β pg/ml	IL-6 pg/ml	IL-10 pg/ml	IL-12p40 pg/ml	TNF pg/ml	IL-8 pg/ml	IP-10 pg/ml	Caspase 3 pg/ml	Caspase 8 ng/ml	Caspase 9 ng/ml
Pericardial fluid	14	D0	Combined	2061 648–3285	0 0–62	15 0–114	9064 8225–10,000	26 21–65	0 0–5	197 100–354	7670 1841–10,434	1414 1013–1646	93 0–313	7 5–16	35 30–73
	5	D0	Prednisolone	1585 533–6363	0 0–149	0 0–109	9277 8533–9969	28 22–47	0 0–3	180 107–469	3725 1512–9987	1435 846–1537	134 0–250	6 5–14	31 24–82
	3	8 h		1436 383–2903	0 0–213	0 0–177	9166 8269–9390	37 25–56	0 0–0	218 142–483	4635 2876–9677	1495 1050–1701	193 0–193	8 6–13	38 26–61
	4	24 h		838 278–2696	0 0–20	0 0–42	8797 8102–9755	24 13–31	0 0–0	265 141–394	1834 1576–2890	1245 865–1335	48 0–137	6 4–9	32 24–42
	9	D0	Placebo	2536 553–3687	0 0–111	18 7–292	8850 7243–10,254	22 17–83	0 0–10	213 99–291	9329 1903–10,855	1348 790–1887	88 0–403	7 5–20	36 30–91
	6	8 h		1347 298–3485	2 0–18	14 8–74	9097 8058–9483	29 11–125	0 0–3	182 96–353	2603 2334–9106	1462 657–1972	72 0–216	10 8–17	41 31–70
	6	24 h		927 482–2619	8 0–124	58 14–185	8695 6377–9394	16 8–46	1 0–18	175 83–299	7067 4180–9957	1758 908–1944	168 0–684	11 4–21	48 32–61
Plasma	12	D0	Combined	28 10–116	0 0–0	0 0–0	23 16–61	4 0–7	0 0–0	33 21–53	23 11–33	82 40–158	0 0–0	0 0–2	24 19–40
	5	D0	Prednisolone	84 28–231	0 0–0	0 0–5	20 13–23	3 0–12	0 0–0	35 28–67	21 16–30	77 26–127	0 0–56	0 0–6	29 23–55
	3	8 h		72 14–102	0 0–0	0 0–5	4 0–15	0 0–8	0 0–0	23 15–25	10 10–25	52 33–72	0 0–0	2 1–4	49 17–55
	3	24 h		37 9–259	0 0–0	0 0–5	6 6–73	0 0–7	0 0–0	19 19–57	28 16–54	49 21–59	0 0–470	2 0–2	25 14–34
	7	D0	Placebo	13 5–118	0 0–0	0 0–0	35 19–88	4 0–7	0 0–0	26 12–55	25 5–72	86 36–305	0 0–0	0 0–0	20 18–34
	5	8 h		16 4–117	0 0–0	0 0–0	49 19–67	6 0–10	0 0–0	22 14–94	20 8–99	68 36–214	0 0–109	2 0–3	23 19–45
	6	24 h		11 3–83	0 0–0	0 0–0	44 17–74	1 0–3	0 0–0	20 17–68	19 16–30	47 24–288	0 0–0	0 0–2	20 15–22
Saliva	12	D0	Combined	17 10–23	3393 949–11,180	28 18–144	11 2–28	15 1–25	18 0–39	15 9–80	295 134–961	1123 167–2316	nd	nd	nd
	5	D0	Prednisolone	18 10–30	1758 980–8567	26 15–663	10 5–29	21 15–57	31 0–48	13 10–47	325 88–1257	1061 201–2420	nd	nd	nd
	3	8 h		17 17–35	341 336–5752	12 5–20	5 1–10	37 4–40	37 10–86	14 7–20	52 32–55	139 45–762	nd	nd	nd
	4	24 h		10 1–36	390 59–3341	11 2–14	6 1–11	28 2–63	23 0–88	12 3–15	44 3–117	92 30–552	nd	nd	nd
	7	D0	Placebo	15 0–23	6473 722–12,416	30 17–146	11 0–29	3 0–22	11 0–39	59 6–210	265 156–486	1184 67–2361	nd	nd	nd
	6	8 h		16 13–17	1399 401–13,354	14 4–56	8 4–30	4 1–37	0 0–2	22 4–110	140 22–234	345 90–1095	nd	nd	nd
	7	24 h		15 11–23	2890 531–7632	26 17–105	10 2–12	5 0–77	0 0–32	26 5–56	163 94–361	376 240–2661	nd	nd	nd

N: number of patient samples available for analysis at specific time point; D0: day 0; nd: not done.

**Fig. 1:**
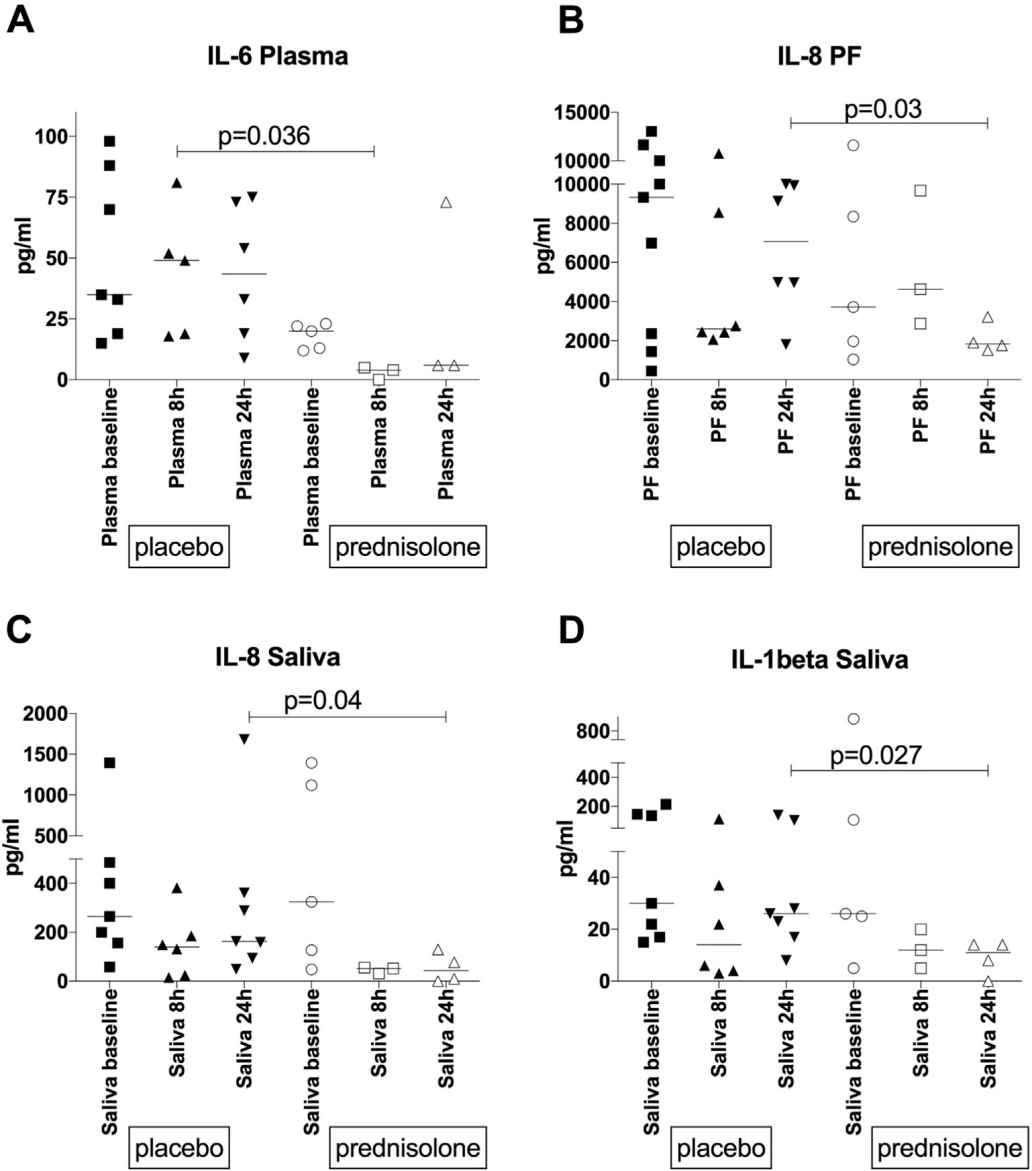
Panel A. Concentration of IL-6 (pg/ml) in plasma at baseline, 8 and 24 h after initiation of prednisolone treatment, which resulted in significant reduction by 8 h (p = 0.036, Mann Whitney test). Concentration of IL-8 (pg/ml) in pericardial fluid (Panel B) and saliva (Panel C) at baseline, 8 and 24 h after initiation of prednisolone treatment, which led to significant decrease by 24 h in both pericardial fluid and saliva (p = 0.03 and 0.04 respectively, Mann Whitney test). Panel D. Concentration of IL-1beta (pg/ml) in saliva at baseline, 8 and 24 h after initiation of prednisolone treatment, leading to significant decrease by 24 h (p = 0.027, Mann Whitney test).

Prednisolone significantly decreased the concentration of IL-6 by 8 h in plasma, compared to the patients who received placebo (Fig. 1A, p = 0.036, Mann Whitney *U* test). In pericardial fluid, prednisolone significantly reduced IL-8 concentrations by 24 h (Fig. 1B, p = 0.03, Mann Whitney *U* test), compared to the placebo treated patients. There was a trend towards decreased IL-1beta concentrations, however significance was not reached due to low numbers (p = 0.06, not shown). Finally in saliva, we found a significant reduction in concentration of both IL-8 and IL-1beta by 24 h of treatment in the prednisolone group (Fig. 1C, p = 0.04, Mann Whitney *U* test; and Fig. 1D, p = 0.027, Mann Whitney *U* test), compared to the placebo group. The concentration of caspase 3, 8 and 9 did not appear to be affected by prednisolone in any of the compartments evaluated.

## 4 Discussion

The largest clinical trial evaluating the effect of adjunctive prednisolone therapy showed no effect on the primary composite of death, cardiac tamponade requiring pericardiocentesis or constrictive pericarditis, however, with respect to the secondary outcomes, the IMPI trial demonstrated that prednisolone reduced the incidence of constrictive pericarditis and the incidence of hospitalisations [3]. Here we hypothesised that the antiinflammatory effects of prednisolone would also manifest in reduced measurable concentrations of inflammatory cytokines and chemokines. Our findings suggest that the beneficial effect of prednisolone is associated with the suppression of inflammatory mediators IL-6, IL-8 and IL-1beta.

These results support our previous findings described during the treatment of tuberculosis associated immune reconstitution inflammatory syndrome (TB-IRIS) with prednisone vs placebo [9], where prednisone reduced the duration of hospitalisation and the number of outpatient therapeutic procedures. At the same time, IL-6, IL-10, IL-12 p40, TNF, IFN-gamma, and IFN-gamma-induced protein-10 (IP-10, CXCL10) concentrations significantly decreased in the serum of prednisone, but not placebo, treated patients [10]. In a separate study we found that adjunct corticosteroid therapy modifies the inflammatory profile of those who develop TB-IRIS, with lower concentrations of IFN-gamma, IP-10, TNF, IL-6, IL-8, IL-10, IL-12p40, and IL-18 [11]. Thus, the beneficial effects of prednisone appear to be mediated via suppression of predominantly proinflammatory cytokine responses of innate immune origin, similar to our current findings.

We reported earlier that cell-death enrichment factors were elevated in pericardial fluid [4] and hypothesised that TB antigen specific activated T cells that enter the pericardium and release INF-gamma die, thus potentially activating the inflammasome pathway resulting in pyroptosis and release of IL-1beta, ultimately leading to more cell death. Here we show a trend towards decreased IL-1beta concentrations in the pericardial fluid of prednisolone treated patients, as well as higher concentrations of caspases 3, 8 and 9 in the pericardial fluid, thereby supporting our hypothesis for compartmentalized inflammation induced apoptosis in the pericardial fluid.

This report is limited by the small sample size therefore the statistical significance of the findings is weak. The small sample size further limited our capacity to analyse the results based on the HIV status of the patients, since it has been described that HIV infection is associated with a lower incidence of pericardial constriction in patients with presumed tuberculous pericarditis [12], and IL-13-secreting CD4 T cells, which are reduced by HIV, regulate fibrogenesis directly, through stimulating collagen synthesis by fibroblasts and indirectly by promoting TGF-beta1 production by macrophages [13]. However, we based our analysis strategy on our previous findings, that showed neither HIV-1 coinfection status, nor CD4 count or pericardial fluid *Mycobacterium tuberculosis* culture result affected the compartmentalized profibrotic immune response with respect to the selected analytes we measured [4]. Additional limitation of this pilot study is that pericardial fluid sampling was restricted within 24 h of initiation of prednisolone therapy due to safety concerns over leaving the pericardial catheter in the pericardial space for a prolonged period of time.

These findings however are of interest because TB remains the leading cause of constrictive pericarditis in Africa. The treatment for chronic pericardial tuberculosis leading to constriction is pericardiectomy, which is associated with high mortality and morbidity but also problematic due to the fact that cardiac surgery is not widely available in Africa [14]. Thus adjunctive immunotherapy that reduces the incidence of constrictive pericarditis, and which could be monitored by measuring inflammatory markers in easily obtainable samples such as plasma or saliva, might be beneficial during early patient follow up in reducing morbidity and possibly even mortality.

## Author contribution

JS, ILR, BMM, RJW, MN and KAW conceived and designed the study. JS, ILR, NP, BMM recruited, sampled and collected data from patients. KAW performed the experiments with input into experimental design from RPL. KAW and RJW analysed the data. KAW, RJW wrote the manuscript, which was revised by all authors.

## Funding

This work was supported by the a South African Medical Research Council Self-Initiated Award; The Francis Crick Institute (FC00110218); The Wellcome Trust (104803, 203135); National Research Foundation of South Africa (96841); European Union TBVAC2020 (643381).

## Conflict of interest

The authors report no relationships that could be construed as a conflict of interest.
